# THBS4/integrin α2 axis mediates BM-MSCs to promote angiogenesis in gastric cancer associated with chronic *Helicobacter pylori* infection

**DOI:** 10.18632/aging.203334

**Published:** 2021-08-14

**Authors:** LingNan He, WeiJun Wang, HuiYing Shi, Chen Jiang, HaiLing Yao, YuRui Zhang, Wei Qian, Rong Lin

**Affiliations:** 1 Department of Gastroenterology, Union Hospital, Tongji Medical College, Huazhong University of Science and Technology, Wuhan 430022, China

**Keywords:** bone marrow-derived mesenchymal stem cells (BM-MSCs), gastric cancer (GC), tumor angiogenesis, THBS4/Integrin α2 axis, PI3K/AKT pathway

## Abstract

Background: BM-MSCs contribute to *Helicobacter pylori (H. pylori)*-induced gastric cancer, but their mechanism is still unclear. The aim of our study was to investigate the specific role and mechanism of BM-MSCs in *H. pylori*-induced gastric cancer.

Main methods: Mice received total bone marrow transplants and were then infected with *H. pylori*. BM-MSCs were extracted and transplanted into the gastric serosal layer of mice chronically infected with *H. pylori*. Hematoxylin and eosin staining, immunohistochemistry staining and immunofluorescence were performed to detect tumor growth and angiogenesis in mouse stomach tissues. Chicken chorioallantoic membrane assays, xenograft tumor models, and human umbilical vein endothelial cell tube formation assays were used for *in vivo* and *in vitro* angiogenesis studies. THBS4 was screened from RNA-seq analysis of gastric tissues of BM-MSCs transplanted into *H. pylori-*infected mice.

Results: BM-MSCs can migrate to the site of chronic mucosal injury and promote tumor angiogenesis associated with chronic *H. pylori* infection. Migration of BM-MSCs to the site of chronic mucosal injury induced the upregulation of THBS4, which was also evident in human gastric cancer and correlated with increased blood vessel formation and worse outcome. The THBS4/integrin α2 axis promoted angiogenesis by facilitating the PI3K/AKT pathway in endothelial cells.

Conclusions: Our results revealed a novel proangiogenic effect of BM-MSCs in the chronic *H. pylori* infection microenvironment, primarily mediated by the THBS4/integrin α2 axis, which activates the PI3K/AKT pathway in endothelial cells and eventually induces the formation of new tumor vessels.

## INTRODUCTION

Gastric cancer (GC) is the fifth most common cancer and the third most common cause of cancer death globally [1]. Many factors are implicated in the carcinogenesis and progression of GC, including the prevalence of *Helicobacter pylori* infection, the main cause of GC [2]. Chronic *H. pylori* infection of the gastric mucosa leads to stepwise progression from atrophic gastritis and intestinal metaplasia and eventually adenocarcinoma [3]. However, the mechanism by which *H. pylori* infection mediates GC progression is incompletely defined.

Recent studies on GC demonstrated that cancer stem cells (CSCs) exist in GC. These cells could originate from bone marrow-derived cells (BMDCs) [4, 5]. Bone marrow-derived mesenchymal stem cells (BM-MSCs) are among the BMDCs that have been shown to be recruited to GC and to promote tumor growth [6–8]. Houghton et al demonstrated that chronic infection of C57BL/6 mice with *Helicobacter* induced an environment conducive to BMDC recruitment, with BM-MSCs being the most likely candidate [9]. Varon demonstrated that infection by *H. pylori* leads to bone marrow (BM) stem cells and particularly BM-MSCs recruitment and homing in the gastric mucosa and that BM-MSCs-reconstituted gastric glands can evolve toward metaplasia and dysplasia [10]. Quante et al demonstrated that a significant percentage of carcinoma-associated fibroblasts (CAFs) in *Helicobacter felis*-infected inflammation-associated GC originate from BM-MSCs, which promote cancer growth and progression [11]. Studies using BM transplantation in mice have shown that BM-MSCs display a remarkable feature called tumor-specific tropism, reflective of their active migration to tumor sites [12]. However, the specific mechanism by which BM-MSCs are involved in the occurrence of *H. pylori*-related GC remains uninvestigated.

Several studies have pointed out that BM-MSCs promote tumor growth by enhancing angiogenesis [13–16]. Nevertheless, whether BM-MSCs participate in the occurrence of GC associated with chronic *H. pylori* infection by promoting tumor angiogenesis is unknown. Since the *in vivo* distinct functional role of BM-MSCs in facilitating blood vessel formation in spontaneous primary gastric tumors associated with chronic *H. pylori* infection remains unexplored, the aim of this research was to investigate the role of BM-MSCs and their specific mechanism in angiogenesis associated with chronic *H. pylori* infection.

## MATERIALS AND METHODS

### *Helicobacter* strain and infection experiments

*H. pylori* strain SS1 was cultured in *H. pylori* fluid nutrient medium (HB8647, Hopebio, QingDao, China) under microaerobic conditions (N2: 85%; O2: 5%; CO2: 10%) at 37° C. *H. pylori* strain SS1 was collected in phosphate buffered saline (PBS) (Gibco, Thermo Fisher, MA, USA), and male BALB/c mice (4-6 weeks of age) were fasted for 6 h and inoculated with the bacterial suspension (1 ~ 2 x10^9^ CFU/mL, 100 μl per mouse) by oral gavage every other day for 3 days. Noninfected control groups were given PBS alone.

### Total bone marrow transplantations (BMT)

BALB/c male mice were lethally irradiated using an X-ray machine. Then, 24 h after irradiation, the mice were injected intravenously (i.v.) with unfractionated BM cells harvested aseptically from age-matched LSL-tdTomato male mice. Following transplantation, mice received antibiotics for 4 weeks in water (enrofloxacin; 0.2 mg/ml). After three months of chronic *H. pylori* infection, engraftment of LSL-tdTomato marrow-derived cells was tracked with immunofluorescence (IF) staining of tdTomato.

### BM-MSC extraction and transplantation

After 3 months of chronic *H. pylori* infection, a GFP fluorescently labeled BM-MSC transplantation model was established. BM-MSCs were obtained by femoral lavage of 4-week-old male GFP transgenic BALB/c mice. Cells were cultured and identified by flow cytometry detection of cell surface antigens, including CD45, CD73, CD105, stem cell antigen-1 (Sca1), and CD11b (antibodies all from BD-Pharmingen, CA, USA) (Supplementary Figure 1). A total of 2x10^6^ collected BM-MSCs from GFP mice were resuspended and washed in PBS and then injected into the serosal layer of the mouse gastric antrum using a 50-μl Hamilton syringe as previously described [17]. After 3 months, the level of engraftment and migration was assessed by confocal microscopy observation of GFP fluorescence in gastric tissues.

### HUVEC extraction and identification

Human umbilical vein endothelial cells (HUVECs) were obtained from the human umbilical vein using type I collagenase Hank’s solution. HUVECs were cultured in endothelial cell medium (ECM) (cat. No. 1001, ScienceCell, CA, USA) containing 25 ml of fetal bovine serum (FBS, Cat. No. 0025), 5 ml of endothelial cell growth supplement (ECGS, Cat. No. 1052) and 5 ml of antibiotic solution (P/S, Cat. No. 0503). Cells were identified by immunofluorescence staining of anti-von Willebrand Factor (VWF) (Bs-0586R, Bioss, Beijing, China) and anti-CD31 (ab9498, Abcam, Cambridge, England) for cell surface antigens (Supplementary Figure 2).

### Cell culture, animals and patient samples

The GC cell line used in our study (SGC7901; SGC) was purchased from the American Type Culture Collection (ATCC). BALB/c mice and BALB/c nude mice were purchased from the Beijing Huafukang Biological Co., Ltd. and Beijing Viton Lihua Biological Co., Ltd. and housed under standard laboratory conditions in the Experimental Animal Center of Tongji Medical College, Huazhong University of Science and Technology. The human sample comes from the Endoscopy Center of Union Hospital. All samples were obtained with the patients’ informed consent, and the samples were processed histologically.

### Conditioned medium (CM) preparation

*H. pylori* strain SS1 was cultured and oscillated in *H. pylori* fluid nutrient medium for 24 h. Bacterial suspension was centrifuged at 4500rpm for 15 min. Then, *H. pylori* supernatant was filtered with a 0.22um filter. Subsequently BM-MSCs were pretreated with *H. pylori* culture supernatant (multiplicity of infection [MOI]=50) for 12 h. The BM-MSC medium was replaced, and the supernatant was collected 48 h later. The supernatant obtained was the conditioned medium from BM-MSCs stimulated by *H. Pylori*.

### *In vitro* co-culture system

*In vitro* co-culture system between HUVECs and SGC cells was established by using a Transwell with six-well plates in which HUVECs were indirectly co-cultured with SGC cells and separated from SGC cells by a semipermeable membrane (pore size of 0.6 μm). After 24 h of co-culture, HUVECs were collected for subsequent experiments such as migration assay and tube formation assay.

### SGC xenograft tumor model

Four-week-old male BALB/c nude mice were housed under standard laboratory conditions. Human GC SGC cells were harvested and resuspended in PBS. A total of 2x10^6^ cells were injected subcutaneously into the right forelimb armpit of BALB/c nude mice. The tumor size was measured every two days. After 21 days, the mice were sacrificed, and tumor growth was evaluated according to weight.

### Chick embryo chorioallantoic membrane (CAM) assay

The CAM assay was performed to evaluate *in vivo* angiogenic activity as previously described [18]. Briefly, 100 fertilized eggs were incubated at 38° C and 36.5-38.5% relative humidity in a forced draught incubator. A small puncture window was made on each 8-day-old fertilized egg. Then, 30 μl of cell suspension was dropped onto a gelatin sponge and added onto the CAM. The eggs were sealed with transparent tape and incubated at 37.8° C and 60% humidity for 4 days. On day 12, the eggs were opened, and the CAM vasculatures were imaged using a stereomicroscope equipped with a digital camera. Angiogenesis was quantified by determining the blood vessel density (percentage of blood vessel area over the whole area under a microscopic field) using the image analysis program IPP 6.0 (Image-Pro Plus, version 6.0, Media Cybernetics).

### Tube formation assay

HUVECs were resuspended to a final concentration of 2x10^5^ cells/ml. μ-Slide Angiogenesis (ibidi, Martin Reid, Germany) was precoated with 10 μl Matrigel (Corning, BD, CA, USA) and then incubated at 37° C and 5% CO2 for 30 min. After Matrigel was solidified, resuspended HUVECs (approximately 1x10^4^ cells) were seeded into a μ-Slide Angiogenesis plate. CM from BM-MSCs or rTHBS4 was added to the μ-Slide Angiogenesis plate. After a 7-h incubation, the tube network was photographed using a phase-contrast microscope. NIH ImageJ with Angiogenesis plugin software was used to assess the tube network and to detect total nodes/tubes/mesh in combination.

### Angiogenesis fluorescence imaging *in vivo*

The AngioSense 750EX Fluorescent Imaging Agent (Perkin Elmer) was injected into the mouse model of GC cell xenografts by tail vein (2 nmol/100 μl per mouse). After 4 h, the probe had accumulated in tumors and enabled imaging of vascularity, perfusion, and vascular permeability at a wavelength of 750 nm. Angiogenesis fluorescence imaging *in vivo* was detected with an *In-Vivo* Xtreme system (Bruker, Baden-Wurttemberg, Germany) and quantified by measuring the tumor uptake of the probe AngioSense 750EX.

### RNA-seq

Total RNA was obtained using Tripure Reagent (Roche Applied Science, IN, USA) following the manufacturer’s instructions. Image analysis, per-cycle base calling and quality score assignment were performed using Illumina Real Time Analysis software (Illumina, CA, USA). First, the magnetic beads with poly(T) probes were hybridized with total RNA to purify mRNA. Then, the mRNA with poly(A) was eluted from the magnetic beads and broken into fragments with a magnesium ion solution. The mRNA fragments were reverse transcribed to cDNA and synthesized to double-stranded cDNA. The amplified cDNA was repaired and purified. Directional RNA-seq libraries resulting from these analyses were sequenced in paired-end format in two different rounds (Illumina HiSeq 2000).

### RNA extraction and gene expression analysis

Total RNA was isolated from triplicate wells under each condition with RNAiso Plus reagent (Code No.: 9109, TAKARA, Japan) and reverse transcribed into cDNA using a reverse transcription system (Code No.: RR036A, TAKARA, Japan) according to the manufacturer’s instructions. The quality and quantity of RNA were measured with a Nanodrop system (Thermo Scientific, MA, USA). Quantitative real-time polymerase chain reaction (qRT-PCR) was performed using TB Green *Premix Ex Taq* (Tli RNaseH Plus) (Code No.: RR420A, TAKARA, Japan). The fold changes in gene expression were obtained after normalization to GAPDH, and 2^−(ΔΔCt)^ was used to calculate the relative abundance of mRNA. The primers used in this study are shown in Supplementary Table 1.

### SDS-PAGE and western blotting analysis

Proteins were extracted in RIPA buffer containing phosphatase and protease inhibitor mixes (Beyotime Biotechnology, China) at 4° C for 10-20 min. The quantity of proteins was measured using a BCA protein concentration determination kit (Beyotime Biotechnology, China). After removing DNA and impurities, proteins were separated by SDS-PAGE (WB2102, BioTides, Beijing, China) and transferred to nitrocellulose membranes. The membrane was blocked using 10% skimmed milk before detection with specific antibodies and HRP-conjugated secondary antibodies in combination with enhanced chemiluminescence. The signal was acquired with Millipore Immobilon Western Chemilum HRP substrate, and the grayscale value was quantified with ImageJ. The primary antibodies used were sheep anti-mouse THBS4 (AF7860-SP, R&D Systems), rabbit anti-AKT (#4691T, CST), and rabbit anti-phospho-AKT (p-AKT) (Ser473) (#4060T, CST).

### Histology, immunohistochemistry (IHC) and IF staining

The tissues were embedded and sectioned. After dewaxing and hydration, the sections were incubated overnight with antibodies against human THBS4 (YT4646, ImmunoWay), human PECAM-1 (ab9498, Abcam), mouse CD31 (AF3628-SP, R&D Systems), mouse NG2 (sc-5389, Santa Cruz), mouse THBS4 (AF7860-SP R&D Systems), and mouse Ki-67 (ab1667, Abcam) and identified with Alexa 488, Alexa 594 or Cy3 secondary antibodies or HRP-conjugated secondary antibodies the next day. The sections were detected by phase-contrast microscopy and confocal microscopy, and raw image data were processed with Image-Pro Plus.

### Cell migration assay

Transwell assays were performed in twenty-four-well plates to determine HUVEC migration. A total of 1000 serum-starved HUVECs cultured in CM from BM-MSCs were plated on the upper chambers of 3.0-μm pore size Transwell inserts, and culture ECM medium containing 20% FBS was introduced to the lower chambers. After incubating the cells for 24 h in a 37° C incubator, unmigrated cells on the top membrane were removed with cotton swabs. The remaining cells were fixed in 4% paraformaldehyde for 15 min, stained with 1% crystal violet for 10 min at room temperature and then washed in PBS 3 times. Images of migratory cells were acquired with an inverted light microscope, and the cells were counted to quantify cell migration.

### Lentivirus construction and transfection

Lentivirus for THBS4 short hairpin RNA (shRNA) and negative control (NC) sequences were designed and constructed by Obio Technology Co. Ltd., Shanghai, China. The shRNA lentivirus was transfected into BM-MSCs using HitransG A (GeneChem, Shanghai, China) according to the manufacturer's protocol. Selection of stably transfected cells was performed with puromycin (8 μg/ml). The targeted THBS4 sequence was as follows: GGATGAGTGCAAATACCAT.

### Statistical analysis

All experiments were repeated at least three times, and the results were expressed as the means ± SD. All statistical analyses were performed using GraphPad Prism software. Student’s t-test or one-way analysis of variance (ANOVA) was used to analyze the data, and the chi-square test was used to analyze differences in other variables, as appropriate. Differences with a P value of ≤0.05 were considered statistically significant for all datasets (*p < 0.05, **p < 0.01, ***p < 0.001).

## RESULTS

### BM-MSCs exacerbate tumorigenicity and angiogenesis in *H. pylori*-induced GC

**Figure 1:**
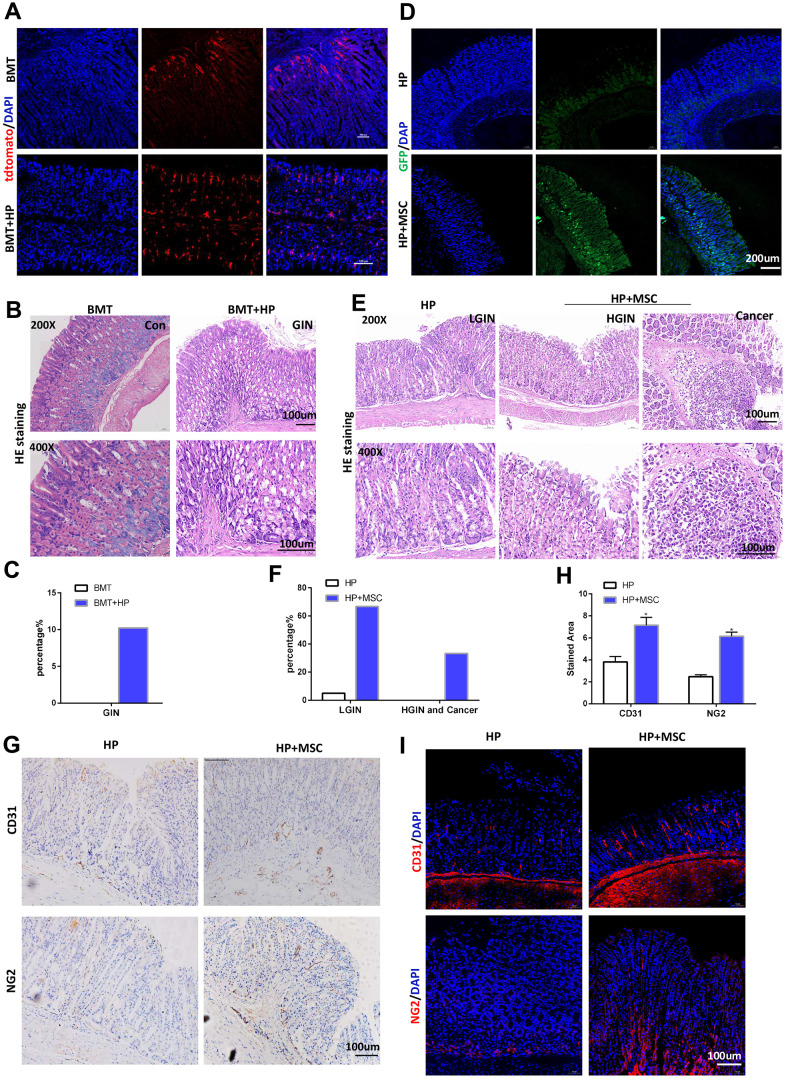
**BM-MSCs promote tumorigenicity and angiogenesis in H. pylori-induced GC.** (**A**) In BMT mice, engraftment of LSL-tdTomato marrow-derived cells in mouse gastric tissues was tracked with tdTomato staining. n= 4. (**B**) Representative gastric mucosa histopathology of gastric intraepithelial neoplasia (GIN) in BMT mice. (**C**) Quantification of GIN in B. (**D**) IF of GFP in *H. pylori*-infected stomachs transplanted with GFP-labeled BM-MSCs. (**E**) Representative gastric mucosa histopathology in *H. pylori*-infected stomachs with (*H. pylori*+ MSC) or without (*H. pylori*) BM-MSC transplantation. n= 30 mice in each group. (**F**) Quantification of dysplasia and GC in D. (**G**) IHC of CD31 and NG2 in *H. pylori* or *H. pylori*+ MSC group. (**H**) Quantification of F. Multiple fields (at least six) from three different mice were analyzed. (**I**) IF of CD31 and NG2 in *H. pylori* or *H. pylori*+ MSC group. Cell nuclei, DAPI; CD31, Cy3; NG2, Cy3. HP: *H. pylori*. MSC: bone marrow-derived mesenchymal stem cells. IF: immunofluorescence. The results are shown as the mean ± SD. *, P < 0.05; **, P < 0.01.

To verify the role of BM-MSCs by depleting or inhibiting intrinsic BM-MSCs, we established a total BMT model and then infected the mice with or without *H. pylori* for 3 months. In BMT mice, engraftment of LSL-tdTomato marrow-derived cells was detectable in the mouse mucosal layer with tdTomato staining at 3 months of *H. pylori* infection. There was no significant migration of tdtomato-expressing BM-MSCs to the mucosal layer in BMT mice without H. pylori infection (Figure 1A). Hematoxylin and eosin (HE) staining revealed intraepithelial neoplasia, but no tumors were detected in BMT mice with H. pylori infection (Figure 1B, 1C). Then, we conducted BM-MSC transplantation into the serosal layer of the mice in the gastric model to allow more BM-MSCs to reach the gastric mucosal layer, which has a magnifying effect to better study the role of BM-MSCs in inducing GC. Mice were infected with *H. pylori* for 3 months. The gastric serosal layer was engrafted with or without BM-MSCs marked by GFP. After 3 months after transplantation, confocal microscopy confirmed that BM-MSCs could be transplanted into the stomach for a long time under chronic *H. pylori* infection and could migrate to the site of chronic mucosal injury (Figure 1D). HE staining revealed that in mice chronically infected with *H. pylori*, transplantation and engraftment of BM-MSCs led to increased dysplasia and to gastric carcinoma (Figure 1E, 1F). Engraftment of BM-MSCs increased the expression of vasculogenic markers, such as CD31 and NG2, in stomach tissues in mice with *H. pylori* infection (Figure 1G–1I). These findings indicate that BM-MSC engraftment alters the tumor microenvironment of GC induced by chronic *H. pylori* infection-related inflammation and contributes to tumor tumorigenicity and angiogenesis.

### BM-MSCs promotes the angiogenesis of GC *in vivo* and the tube formation and migration of HUVECs *in vitro* following *H. pylori* stimulation

**Figure 2:**
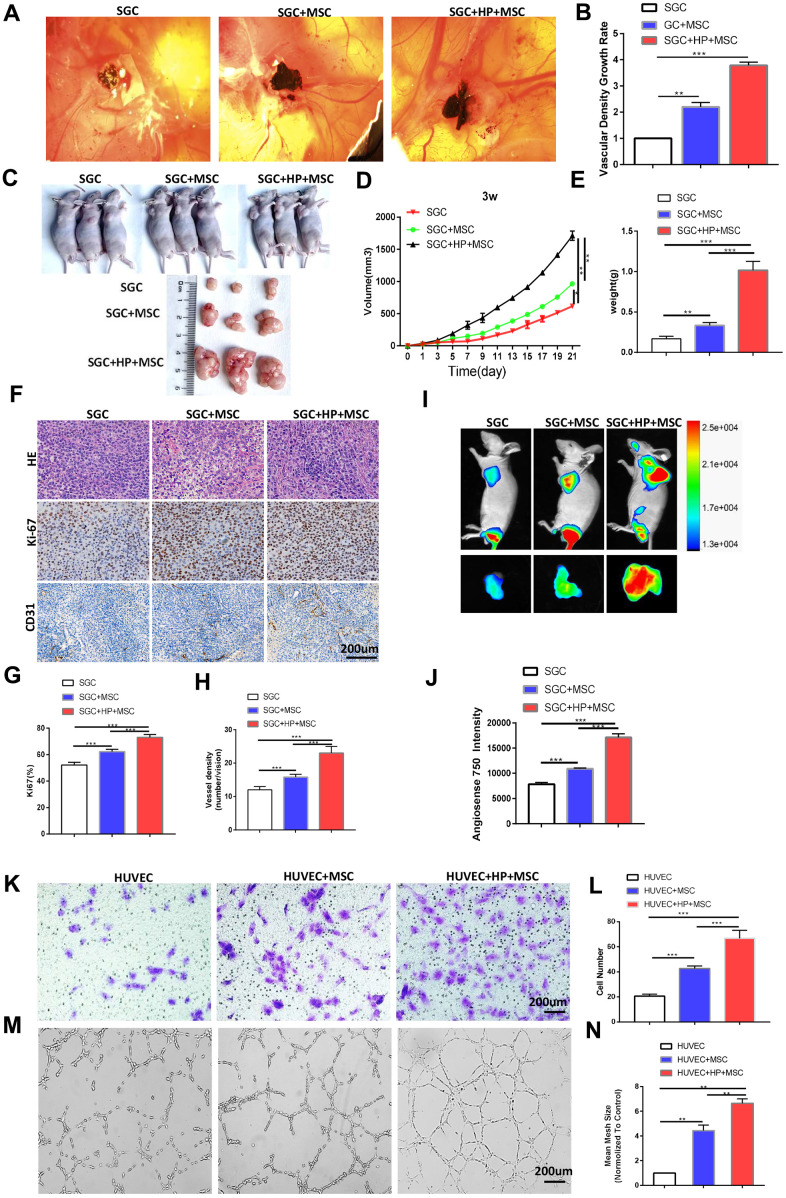
**BM-MSCs promote the angiogenesis of GC *in vivo* and the tube formation and migration of HUVECs *in vitro* following H. pylori stimulation.** (**A**) Representative images of the blood vessels of SGC co-injected with or without BM-MSCs or *H. pylori* -pretreated BM-MSCs in the CAM assay over 4 days. n= 10 eggs in each group. (**B**) Quantification of the vascular density growth rate in the CAM assay. (**C**, **D**) SGC cells mixed with or without BM-MSCs or *H. pylori*-pretreated BM-MSCs were injected into BALB/c nude mice, and tumor volumes were monitored every two days. n= 3 mice in control group and 5 mice per group in other groups (**E**) Average tumor weights from nude mice. (**F**) Representative images of H&E staining and Ki-67 and CD31 (PECAM1) IHC staining of tumors. (**G**, **H**) Quantification of Ki-67 and CD31. (**I**) Angiogenesis fluorescent agent AngioSense 750EX in tumors. (**J**) Quantification of I. (**K**) The migration ability of HUVECs was evaluated using a Transwell assay. n= 3 wells per group. (**L**) Quantification of K. (**M**) *In vitro* tube formation to investigate the effect of CM from BM-MSCs on HUVEC tube formation. n= 5 wells per group. (**N**) Quantification of M.

To explore the specific role of BM-MSCs in the angiogenesis of chronic *H. pylori*-related GC, we pretreated BM-MSCs with *H. pylori* culture supernatant (multiplicity of infection [MOI]=50). We performed a CAM assay on the SGC cells, which has emerged as a standard for *in vivo* angiogenesis assessment [18]. BM-MSCs and SGC cells were mixed and injected into CAMs to detect their role in tumor angiogenesis. Compared with the control, BM-MSCs significantly increased the vascular density of cancer xenografts on the CAM (Figure 2A, 2B). In addition, we subcutaneously transplanted SGC cells mixed with BM-MSCs into BALB/c nude mice. As shown in Figure 2C–2E, SGC cells mixed with BM-MSCs formed subcutaneous tumors with a larger volume and weight than SGC cells alone. Ki-67 IHC staining supported that BM-MSCs play a stimulatory role in gastric tumor growth. CD31 IHC staining revealed that the microvessels in tumors formed by SGC cells mixed with BM-MSCs were more developed (Figure 2F–2H). Tumor vascular permeability fluorescence imaging *in vivo* demonstrated that BM-MSCs significantly increased the degree of tumor neovascularization in SGC-induced tumor xenografts (Figure 2I, 2J).

We established an *in vitro* co-culture system, in which HUVECs were indirectly cocultured with SGC cells and separated from SGC cells by a semipermeable membrane. Transwell assays revealed that treatment with CM from BM-MSCs stimulated by *H. pylori* promoted the migration of HUVECs (Figure 2K, 2L). Meanwhile, HUVEC tube formation was evaluated, and CM from BM-MSCs stimulated by *H. pylori* was proven to promote HUVECs to form a spider web-like microvascular network structure (Figure 2M, 2N).

### BM-MSC engraftment leads to THBS4 overexpression during *H. pylori* infection

**Figure 3:**
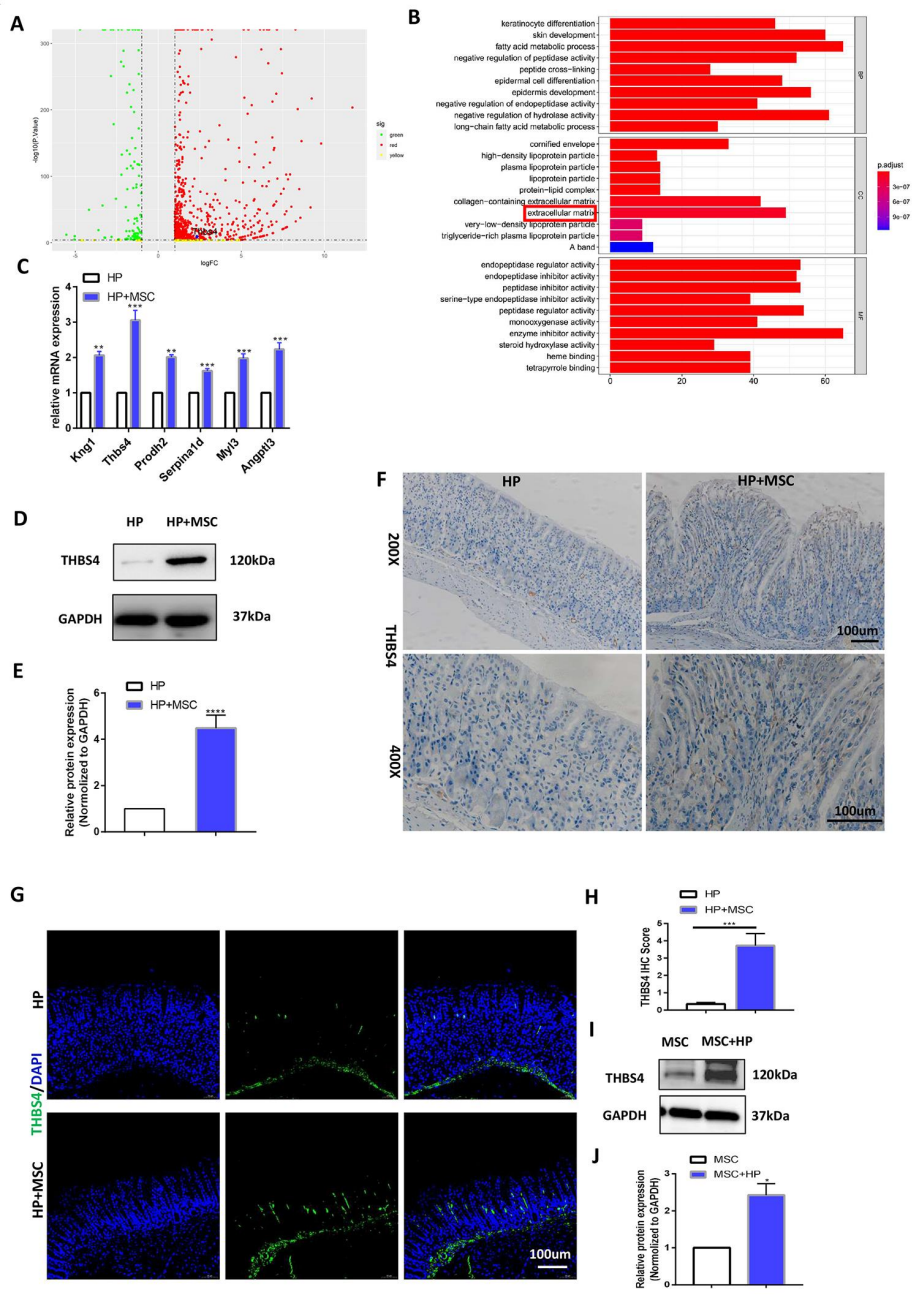
**BM-MSC engraftment leads to THBS4 overexpression during H. pylori infection.** (**A**) Volcano plot presentation of differentially expressed genes of gastric tissues in *H. pylori* versus *H. pylori*+ MSCs mice. (**B**) Enrichment analysis of differential genes. (**C**) RT-PCR verified the expression of differential genes associated with angiogenesis. THBS4 was the most highly expressed gene. n= 10 mice in each group. (**D**, **E**) Western blot analysis showed that BM-MSC transplantation led to THBS4 overexpression. n= 3 mice in each group. (**F**, **G**) IHC (**F**) and IF (**G**) of THBS4 in *H. pylori*+ MSCs or *H. pylori* mice. n= 3 mice per group Cell nuclei, DAPI. THBS4, FITC. Magnification: × 200 and × 400. (**H**) Quantification of F. (**I**, **J**) Western blot analysis of THBS4 revealed that *H. pylori* pretreatment increased the protein expression of THBS4 in BM-MSCs. n= 3 wells per group.

To explore the specific mechanism by which BM-MSCs promote chronic *H. pylori* infection-related GC angiogenesis, we performed RNA-seq analysis to screen for differentially expressed genes. The results showed that BM-MSC transplantation caused the upregulation of many genes (Figure 3A). Genes related to angiogenesis were obtained through enrichment (Gene Ontology; GO) analysis (Figure 3B). The differentially expressed genes were further verified in gastric tissues at the mRNA level. The results of qRT-PCR experiments demonstrated that compared with that in the chronic *H. pylori* infection group, the expression of the Kng1, THBS4, Prodh2, Serpina1d, Myl3 and Angptl3 genes was increased significantly by BM-MSC transplantation (Figure 3C). THBS4 was the most highly expressed gene, and Western blot analysis further indicated THBS4 protein overexpression after BM-MSC transplantation (Figure 3D, 3E). Subsequently, the same result was detected by IHC and IF of THBS4 in stomach tissues (Figure 3F–3H). The effect of *H. pylori* on THBS4 protein expression in BM-MSCs was further tested, and it was found that *H. pylori* pretreatment increased the expression of THBS4 protein in BM-MSCs (Figure 3I, 3J).

### Overexpression of THBS4 is correlated with vessel density in human GC tissues and predicts a poor prognosis in GC patients

**Figure 4:**
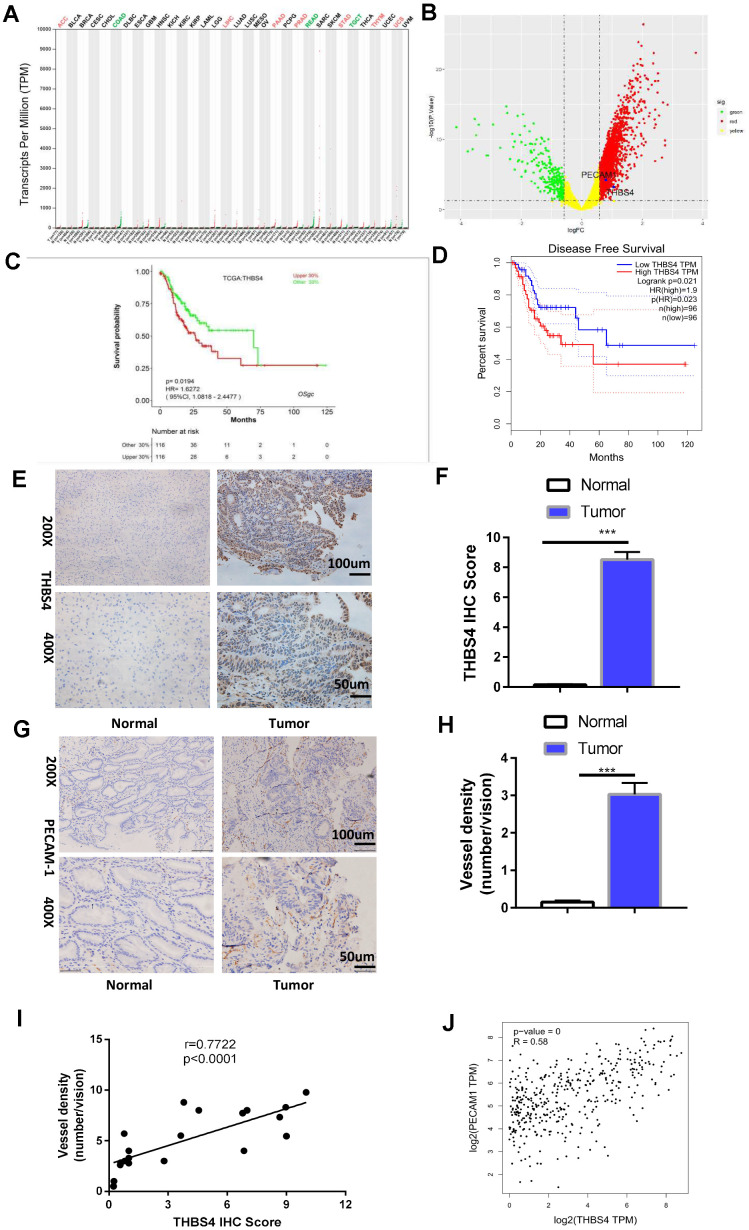
**Overexpression of THBS4 is correlated with vessel density in human GC tissues and predicts a poor prognosis.** (**A**) The expression of THBS4 in a pan-cancer dataset. The red and green labels at the top represent high and low expression in certain tumor types, respectively. (**B**) Volcano plot revealed upregulation of THBS4 mRNA expression in GC compared with normal tissue. (**C**, **D**) The overall survival and disease-free survival analyses of THBS4 were plotted for patients with GC in TCGA. (**E**) Representative IHC staining with THBS4 antibody in GC and adjacent nontumor tissues. n= 5 samples per group. (**F**) Quantification of E. (**G**) Representative IHC staining with PECAM1 antibody in GC and adjacent nontumor tissues. n= 5 samples per group. (**H**) Quantification of G. (**I**) Correlation analysis of THBS4 and vessel density showed that THBS4 was positively correlated with vessel density in GC. (**J**) Validation of the positive correlation between THBS4 and PECAM1 in GEPIA.

THBS4 is a member of the extracellular calcium-binding protein family and is involved in cell adhesion and migration [19]. Bioinformatic analysis of THBS4 expression profiles through The Cancer Genome Atlas (TCGA) indicated that THBS4 was significantly increased in the majority of tumor types (Figure 4A). Notably, THBS4 was expressed at significantly higher levels in gastric carcinoma than in noncancerous stomach tissues (Figure 4B). Furthermore, TCGA database analysis showed that THBS4 expression was negatively correlated with the survival of GC patients (Figure 4C, 4D). We then detected THBS4 protein expression in *H. pylori* (+) GC tissues and corresponding *H. pylori* (+) nontumor tissues by IHC. THBS4 was located in the tumor stroma in gastric adenocarcinoma. In adjacent nontumor tissues, no positive expression was detected (Figure 4E, 4F). THBS4 mRNA expression has been demonstrated to be associated with GC progression [20]. The IHC staining of PECAM1 demonstrated that the blood vessel density in *H. pylori* (+) tumor tissues was significantly higher than that in *H. pylori* (+) nontumor tissues (Figure 4G, 4H). Further analysis revealed that the expression of interstitial THBS4 was positively correlated with the blood vessel density in tumor tissues (Figure 4I). Similarly, bioinformatic analyses of the correlation between THBS4 and PECAM1 were performed through Gene Expression Profiling Interactive Analysis (GEPIA) (Figure 4J). These findings confirmed that THBS4 may play a vital role in the angiogenesis of GC.

### THBS4 mediates BM-MSC-induced angiogenesis in GC

**Figure 5:**
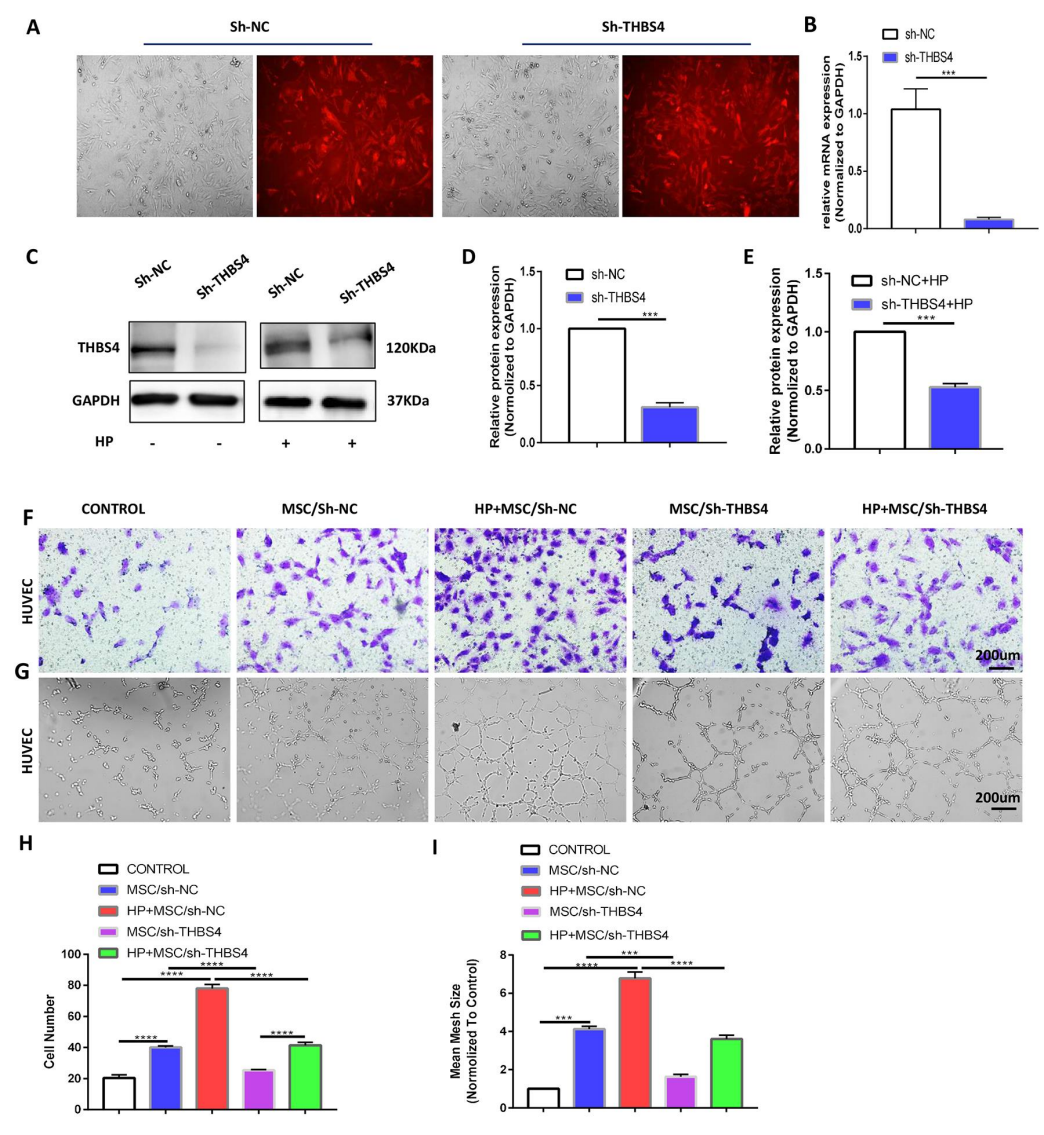
**THBS4 mediates BM-MSCs to promote the migration and tube formation of HUVECs *in vitro*.** (**A**) Transfection of recombinant lentiviral vectors into BM-MSCs. (**B**–**E**) qRT-PCR and Western blot analysis of THBS4 expression in BM-MSCs of both experiments transfected as indicated. n= 3 wells per group. (**F**, **H**) Transwell assays and quantification of migrated cells showed that THBS4 knockdown in BM-MSCs inhibited their ability to promote HUVEC migration. n= 3 wells in each group. (**G**, **I**) Knockdown of THBS4 in BM-MSCs alleviated its ability to promote HUVEC tube formation. n= 5 wells per group.

**Figure 6:**
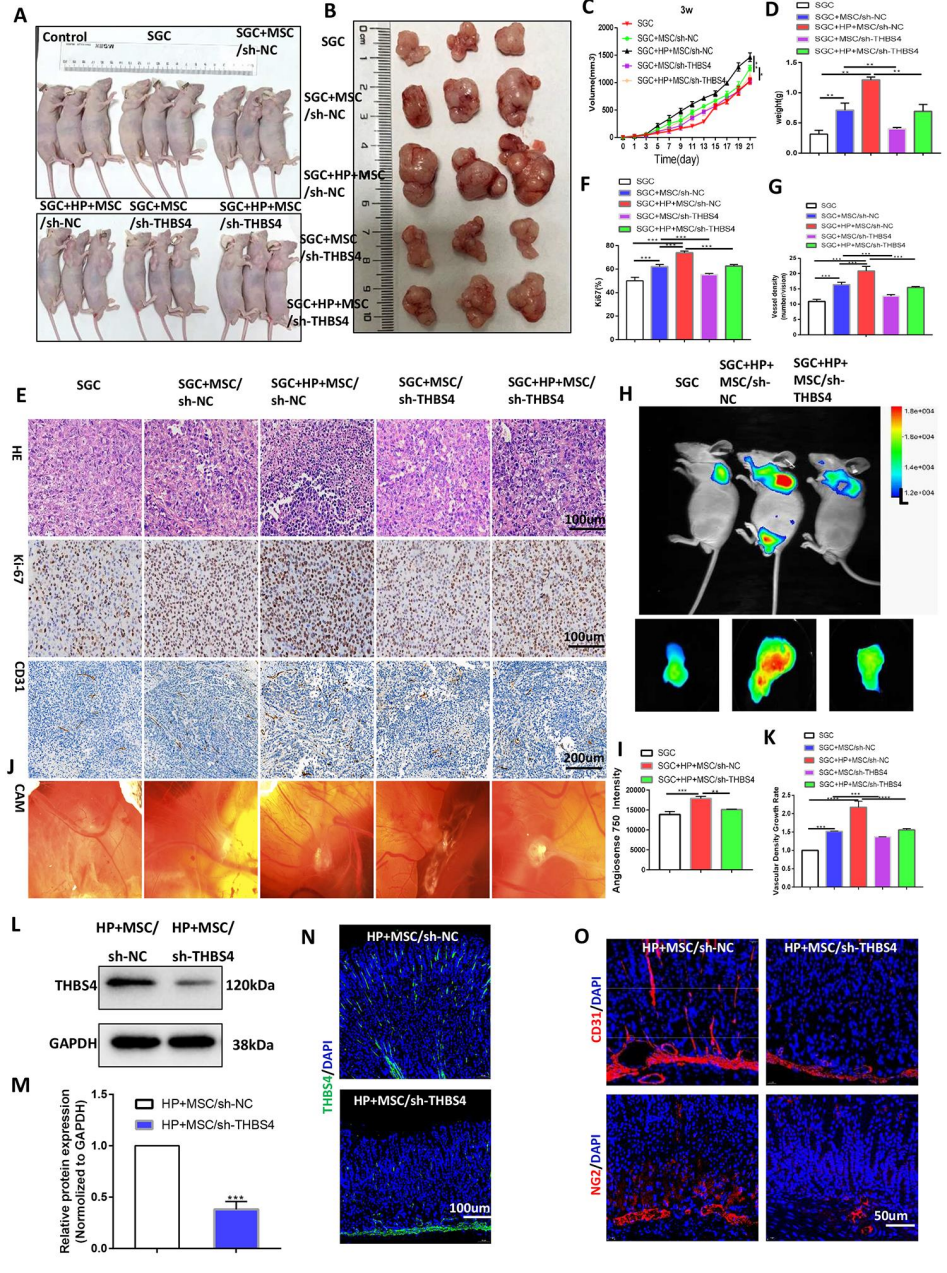
**THBS4 mediates BM-MSC-induced promotion of GC angiogenesis *in vivo*.** (**A**, **B**) Four-week-old nude male mice were injected with either SGC cells only or SGC and BM-MSCs transfected with recombinant lentiviral vectors. Three weeks following injection, the mice were euthanized, and the tumors were analyzed. n= 3 mice in control group and 5 mice per group in other groups. (**C**) Growth curve of tumors described in A. (**D**) Tumor weight of injected tumors at end-point. (**E**) H&E staining and Ki-67 and CD31 immunostaining of tumors described in A. (**F**, **G**) Quantification of staining presented in E performed with Image Plus. (**H**, **I**) Following AngioSense 750EX injection, tumor vascular permeability fluorescence imaging *in vivo* was performed to detect the neovascularization density of tumors in A. (**J**, **K**) Representative images and quantification of the CAM assay. n= 5 eggs in each group. (**L, M**) Western blot analysis of THBS4 in gastric tissues after NC BM-MSC or THBS4-knockdown BM-MSC transplantation. n= 3 mice in each group. (**N**) IF of THBS4 in gastric tissues after NC BM-MSC or THBS4-knockdown BM-MSC transplantation. (**O**) IF of CD31 and NG2 in gastric tissues in NC BM-MSC-transplanted mice and THBS4-knockdown BM-MSC-transplanted mice. n= 10 mice in each group.

To investigate the biological behaviors of THBS4 in BM-MSCs promoting angiogenesis, we successfully constructed lentiviral vectors (sh-THBS4 and sh-NC) (Figure 5A). Real-time PCR and Western blotting were performed to detect whether the expression level of THBS4 changed in BM-MSCs (Figure 5B–5E). In a co-culture system as previously described, Transwell assays and tube formation assays revealed that downregulation of THBS4 reduced the migratory ability and tube formation induced by CM from *H. pylori*-pretreated BM-MSCs (Figure 5F–5I). The results from the xenograft tumor model revealed that injection of *H. pylori*-pretreated BM-MSCs dramatically increased the growth of GC subcutaneous tumors, and these effects were also notably inhibited when THBS4 was silenced (Figure 6A–6D). The results of IHC staining against CD31 and Ki-67 showed that THBS4 silencing in BM-MSCs was related to microvessel density and GC proliferation reduction in subcutaneous tumors (Figure 6E–6G). Tumor vascular permeability fluorescence imaging *in vivo* demonstrated that THBS4-knockdown *H. pylori*-pretreated BM-MSCs demonstrated a weakened neovascularization density compared with *H. pylori*-pretreated NC BM-MSCs (Figure 6H, 6I). Moreover, *H. pylori*-pretreated BM-MSCs strongly accelerated GC angiogenesis in the CAM assay, while these effects were dramatically inhibited when THBS4 was silenced (Figure 6J, 6K). We then transplanted NC BM-MSCs and THBS4-knockdown BM-MSCs into the mouse gastric serosa layer with chronic *H. pylori* infection. Western blotting and IF of THBS4 confirmed that knockdown of THBS4 in transplanted BM-MSCs lead to decreased THBS4 expression of gastric tissues *in vivo* (Figure 6L–6N). IF of CD31 and NG2 revealed that NC BM-MSCs were more efficient in inducing angiogenesis (Figure 6O).

### Integrin α2 mediates the effects of paracrine THBS4 signaling

**Figure 7:**
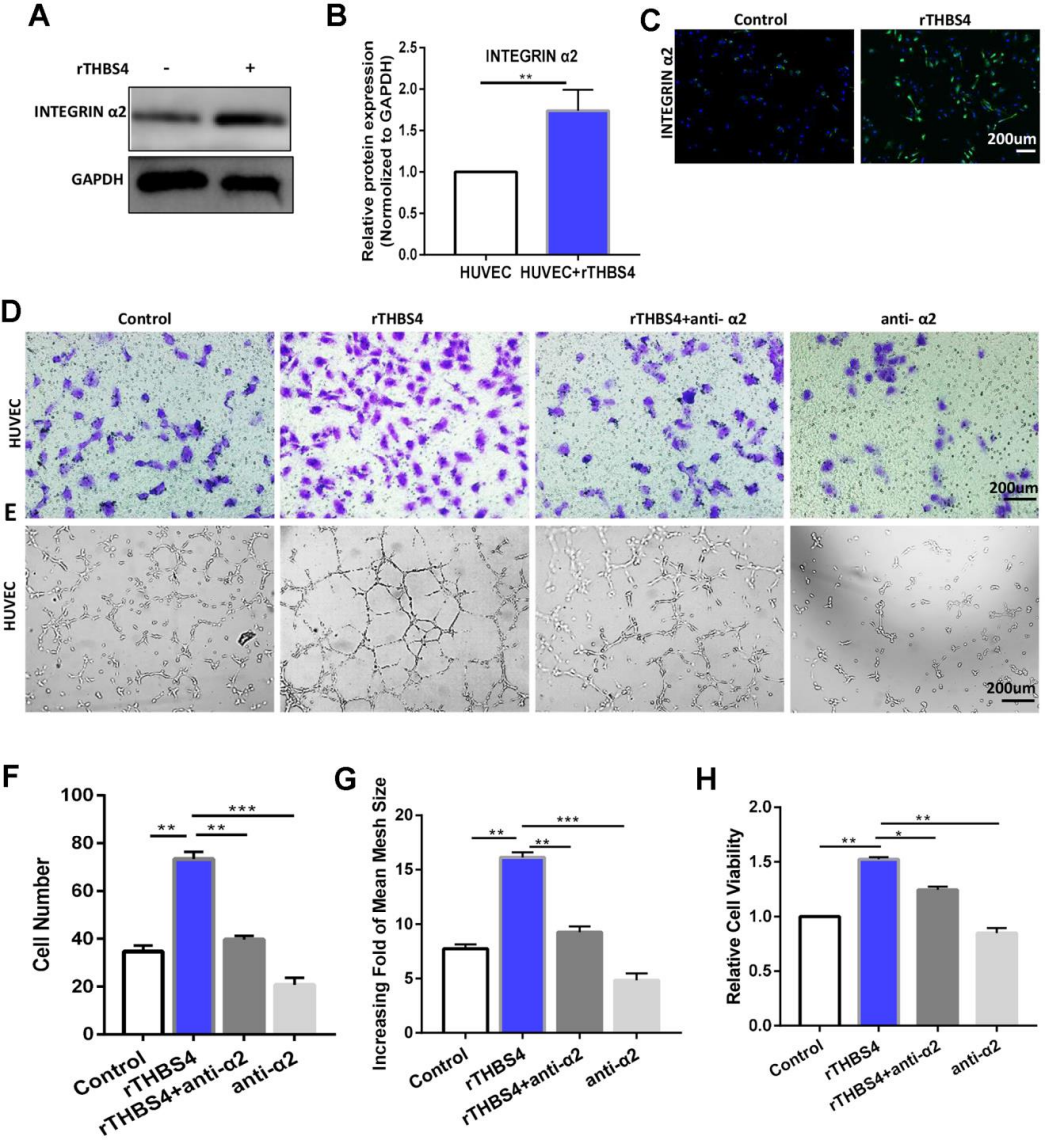
**Integrin α2 mediates the effect of paracrine THBS4 signaling on the migration and tube formation of HUVECs.** (**A**–**C**) Western blotting and IF showed that integrin α2 in HUVECs was significantly increased by rTHBS4. n= 3 wells per group. (**D**) Representative images of the migration assay after rTHBS4, rTHBS4 + anti- α2 or anti- α2 treatments in HUVECs. n= three independent experiments. (**E**) Representative images of the tube formation assay after rTHBS4, rTHBS4 + anti-α2 or anti-α2 treatments in HUVECs. n= 3 wells in each group. (**F**) Quantification of D. (**G**) Quantification of E. (**H**) CCK-8 assay to detect HUVEC proliferation after rTHBS4, rTHBS4 + anti-α2 or anti-α2 treatments. n= three independent experiments.

Integrin α2 was identified as a receptor of THBS4 involved in the regulation of endothelial cell migration [21, 22]. To test whether integrin α2 contributes to promoting angiogenesis in response to rTHBS4, we cultured HUVECs for 48 h in the presence of 10 ng/ml rTHBS4. Western blotting and IF showed that integrin α2 in HUVECs was significantly increased after rTHBS4 treatment (Figure 7A–7C). Furthermore, the molecular mechanisms by which the integrinα2 receptor participates in paracrine THBS4 signaling were investigated using an α2-specific antibody. The α2-specific antibody was able to alleviate the rTHBS4-induced migration and tube formation capacities of HUVECs (Figure 7D–7G). Additionally, the α2-specific antibody abrogated rTHBS4-induced proliferation in HUVECs (Figure 7H).

### Targeting the PI3K/AKT pathway abrogates THBS4/integrin α2 axis-induced promotion of angiogenesis

**Figure 8:**
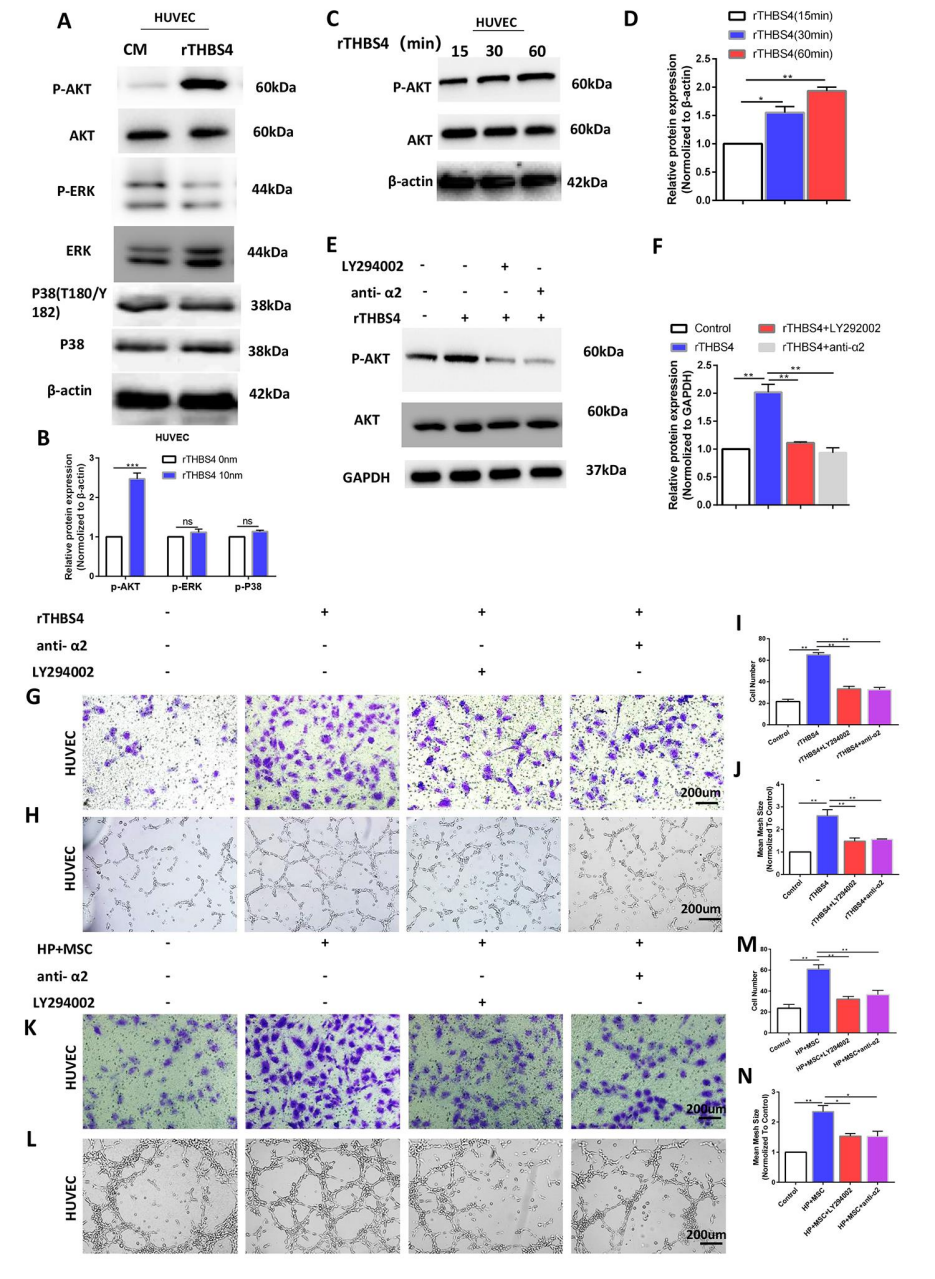
**Activation of AKT by THBS4/integrin α2 axis in HUVECs.** (**A**, **B**) rTHBS4 induced the phosphorylation of AKT in HUVECs. n= three independent experiments. The HUVECs were treated with and without rTHBS4 for 1h and the whole cell lysates were analyzed by WB for levels of downstream kinases. (**C**, **D**) p-AKT activation occurred in a time-dependent manner in rTHBS4-treated cells. n= three independent experiments. (**E**, **F**) The HUVECs were treated with rTHBS4 for 1h in the presence of integrin α2-specific antibody or PI3K inhibitor LY294002. Western blot analysis showed that rTHBS4 increased Akt phosphorylation in HUVECs, while this effect was alleviated by blocking integrin α2 or inhibiting Akt. n= three independent experiments. (**G**) Transwell migration assay to investigate the effect of PI3K inhibition and integrin α2 blockade on HUVEC migration after treatment with rTHBS4. n= 3 wells per group. (**H**) *In vitro* tube formation to investigate the effect of PI3K inhibition and blocking integrin α2 on HUVEC tube formation. n= 3 wells per group. (**I**) Quantification of G. (**J**) Quantification of H. (**K**, **L**) The CM from BM-MSCs stimulated by *H. Pylori* was applied to HUVECs in the presence or absence of integrin α2-specific antibody or PI3K inhibitor LY294002 for Transwell migration assay and tube formation assay. n= 3 wells per group. (**M**) Quantification of K. (**N**) Quantification of L.

The THBS4/integrin α2 axis is known to activate a number of downstream signaling molecules, including PI3K/AKT, ERK, and P38 [23–26]. Then, we evaluated these downstream signaling molecules of the THBS4/integrin α2 axis in HUVECs. The results demonstrated that the p-AKT level was positively correlated with rTHBS4 treatment (Figure 8A, 8B). HUVECs were treated with rTHBS4 for 15–60 min to assess the levels of p-Akt to further verify the results with activation of Akt. The results indicated that p-AKT activation occurred in a time-dependent manner with rTHBS4 treatment (Figure 8C, 8D). Next, we investigated the role and requirement of Akt signaling in rTHBS4 facilitating the migration and tube formation of HUVECs. rTHBS4 treatment enhanced Akt phosphorylation, while this effect was dramatically restrained in the presence of an integrin α2-specific antibody or the PI3K inhibitor LY294002 (Figure 8E, 8F). In addition, the promoting effects on HUVEC migration and tube formation mediated by the rTHBS4/integrin α2 axis were partly counteracted by PI3K inhibition (Figure 8G–8J). Furthermore, we aspired to investigate whether angiogenic capacity of HUVECs was enhanced via integrin α2/Akt in response to THBS4 derived from BM-MSCs. The results demonstrated that the angiogenic capacity of HUVECs was enhanced after treatment of CM from BM-MSCs stimulated by *H. pylori*, while this effect was impaired by anti-α2 or Akt signaling inhibition (Figure 8K–8N). Thus, the THBS4/integrin α2 axis accelerates angiogenesis by activating PI3K/AKT signaling.

## DISCUSSION

Our results show for the first time that BM-MSCs participate in angiogenesis in mice with GC associated with chronic *H. pylori* infection of the gastric mucosa. Our results demonstrated that *H. pylori* induces BM-MSC recruitment and THBS4 production, which induces PI3K/AKT signaling activation in vascular endothelial cells and contributes to *H. pylori*-induced GC angiogenesis. The results revealed the specific role of BM-MSCs in promoting angiogenesis in the occurrence of *H. pylori*-related GC.

Chronic gastric inflammation, which develops as a consequence of *H. pylori*, leads to repetitive injury repair over time, resulting in hyperproliferation, an increased rate of mitotic error, and progression to adenocarcinoma. Recent studies have suggested that in response to chronic *H. pylori* infection, BM-derived cells, especially BM-MSCs, can home to and repopulate the gastric mucosa and contribute over time to etaplasia, dysplasia, and cancer [9, 11]. Nevertheless, the specific role and mechanism of BM-MSCs in the occurrence of GC are unknown. Several studies have pointed to BM-MSCs as a dominant stromal component within the tumor microenvironment that contribute in many ways to tumor progression; these mechanisms include extracellular matrix remodeling, metastasis, and angiogenesis [13, 27, 28]. In the present study, in mice with BMT, engraftment of LSL-tdTomato marrow-derived cells was detectable in mouse gastric tissue after 3 months of *H. pylori* infection. We then showed that transplanted BM-MSCs could migrate to the site of chronic mucosal injury under chronic *H. pylori* infection. Furthermore, engraftment of BM-MSCs led to dysplasia and gastric carcinoma in mice with chronic *H. pylori* infection, and vasculogenic markers were detected in mucosa and submucosa gastric tissues. Thus, the precise effects of BM-MSCs on tumor growth and angiogenesis and the mechanism underlying these effects should be elucidated.

We further found that *H. pylori*-pretreated BM-MSCs significantly promoted vessel formation in GC in the CAM and xenograft tumor models. The oncogenicity and blood vessel formation of GC cells were enhanced by co-implantation with *H. pylori*-pretreated BM-MSCs. Thus, BM-MSCs may affect angiogenesis of the GC tumor microenvironment. Therefore, more studies are necessary to examine whether BM-MSCs harbor this property in endothelial cells. In addition, in *in vitro* experiments, we observed that BM-MSCs promote the angiogenic potential of HUVECs *in vitro*. It appears that the ability of BM-MSCs to promote angiogenesis increases after *H. pylori* treatment.

It has been shown that paracrine factors secreted by BM-MSCs may be involved in tumor angiogenesis [29–31]. A better understanding of the molecular mechanisms by which BM-MSCs promote angiogenesis is important. RNA-seq and GO enrichment analysis revealed that THBS4 may be involved in BM-MSC-mediated promotion of angiogenesis in GC associated with chronic *H. pylori* infection. We also found that THBS4 expression in BM-MSCs increased with *H. pylori* intervention. THBS4 belongs to a group of secreted extracellular matrix proteins and is involved in wound healing and tissue remodeling [32, 33]. Tissue remodeling, inflammation, and angiogenesis often occur simultaneously in the tumor microenvironment and are regulated by the same stimuli. THBS4 was reported to be expressed in some types of solid cancers [34, 35]. The emerging role of THBS4 in tumor-stroma interactions, particularly in GC [34], suggest that it may play an important role in the tumor microenvironment [36]. THBS4 was reported to contribute to the proliferation and metastasis of GC *in vitro* [37]. Muppala S recently reported that THBS4 affects the functions of microvascular endothelial cells and promotes angiogenesis [21, 38] in EMT6 mouse breast cancer cells. What role does THBS4 expressed by BM-MSCs play in *H. pylori*-related GC angiogenesis? Our data indicated that THBS4 derived from BM-MSCs plays a facilitative role in regulating tumor angiogenesis in *H. pylori*-related GC *in vitro* and *in vivo*. These findings suggest that BM-MSCs may stimulate GC angiogenesis via THBS4.

THBS4 is known as a secreted extracellular matrix protein that exerts its effect in multiple ways: by binding to its extracellular ligands and receptors [21, 38, 39], by initiating intracellular signaling [26, 33, 40] and by transiting through secretory pathways [41]. Moreover, we found that integrin α2 expression in HUVECs was significantly increased after rTHBS4 treatment. Integrin α2 was implicated in endothelial cell migration and tube formation mediated by THBS4 using an α2-specific antibody. The THBS4/integrin α2 axis is known to activate a number of downstream signaling molecules, such as PI3K/Akt [25], ERK [26], and p38 [33, 40]. Thus, we further explored whether downstream intracellular signaling participates in THBS4/integrin α2 axis-induced migration and tube formation in HUVECs. We observed that AKT activation is essential for the BM-MSC-derived THBS4-induced migration and tube formation of HUVECs. Sustained endothelial Akt activation causes increased blood vessel size and chronic vascular permeability, which induces the formation of structurally and functionally abnormal blood vessels that recapitulate the aberrations of tumor vessels [42, 43]. Collectively, these results led us to propose a model whereby BM-MSC-derived THBS4 interacts with its receptor integrin α2 to activate the PI3K-Akt pathway, which mediates the migration and tube formation of endothelial cells.

In this study, the association of BM-MSC-derived THBS4 with the angiogenesis of GC associated with *H. pylori* infection was observed *in vivo*, in xenograft tumor models, in CAM assays, and in cultured HUVECs. The THBS4/integrin α2 axis promotes the migration and tube formation of endothelial cells by facilitating the PI3K/AKT pathway, which induces the formation of new tumor vessels in the *H. pylori* infection microenvironment. Insight into the role of BM-MSC-derived THBS4 in angiogenesis may provide a therapeutic target to inhibit angiogenesis in *H. pylori* infection-related GC.

## CONCLUSIONS

Our results provide the basis for the view that BM-MSC-derived THBS4 may play an important role in angiogenesis in GC associated with chronic *H. pylori* infection, suggesting that the THBS4/integrin α2 axis may be a new prognostic and therapeutic biomarker for GC associated with chronic *H. pylori* infection.

### Ethical considerations

All procedures followed were in accordance with the ethical standards of the Ethical Committee of Tongji Medical College, Huazhong University of Science and Technology and with the Helsinki Declaration of 1964 and later versions. The IACUC Number is 2451. All human samples were obtained with the patients’ written informed consent. All institutional and national guidelines for the care and use of laboratory animals were followed.

### Availability of data and materials

Please contact the corresponding author for all data requests.

## Supplementary Material

Supplementary Figures

Supplementary Table 1

## References

[1] Smyth EC, Nilsson M, Grabsch HI, van Grieken NC, Lordick F. Gastric cancer. Lancet. 2020; 396:635–48. 10.1016/S0140-6736(20)31288-5 32861308

[2] Hooi JK, Lai WY, Ng WK, Suen MM, Underwood FE, Tanyingoh D, Malfertheiner P, Graham DY, Wong VW, Wu JC, Chan FK, Sung JJ, Kaplan GG, Ng SC. Global Prevalence of Helicobacter pylori Infection: Systematic Review and Meta-Analysis. Gastroenterology. 2017; 153:420–29. 10.1053/j.gastro.2017.04.022 28456631

[3] Nagtegaal ID, Odze RD, Klimstra D, Paradis V, Rugge M, Schirmacher P, Washington KM, Carneiro F, Cree IA, and WHO Classification of Tumours Editorial Board. The 2019 WHO classification of tumours of the digestive system. Histopathology. 2020; 76:182–88. 10.1111/his.13975 31433515 PMC7003895

[4] Lizárraga-Verdugo E, Avendaño-Félix M, Bermúdez M, Ramos-Payán R, Pérez-Plasencia C, Aguilar-Medina M. Cancer Stem Cells and Its Role in Angiogenesis and Vasculogenic Mimicry in Gastrointestinal Cancers. Front Oncol. 2020; 10:413. 10.3389/fonc.2020.00413 32296643 PMC7136521

[5] Song S, Chen Q, Li Y, Lei G, Scott A, Huo L, Li CY, Estrella JS, Correa A, Pizzi MP, Ma L, Jin J, Liu B, et al. Targeting cancer stem cells with a pan-BCL-2 inhibitor in preclinical and clinical settings in patients with gastroesophageal carcinoma. Gut. 2021. [Epub ahead of print]. 10.1136/gutjnl-2020-321175 33487592 PMC9720890

[6] Ma X, Chen J, Liu J, Xu B, Liang X, Yang X, Feng Y, Liang X, Liu J. IL-8/CXCR2 mediates tropism of human bone marrow-derived mesenchymal stem cells toward CD133^+^ /CD44^+^ Colon cancer stem cells. J Cell Physiol. 2021; 236:3114–28. 10.1002/jcp.30080 33078417

[7] Wang M, Zhao X, Qiu R, Gong Z, Huang F, Yu W, Shen B, Sha X, Dong H, Huang J, Wang L, Zhu W, Xu W. Lymph node metastasis-derived gastric cancer cells educate bone marrow-derived mesenchymal stem cells via YAP signaling activation by exosomal Wnt5a. Oncogene. 2021; 40:2296–308. 10.1038/s41388-021-01722-8 33654199 PMC7994201

[8] Ikeda T, Nishita M, Hoshi K, Honda T, Kakeji Y, Minami Y. Mesenchymal stem cell-derived CXCL16 promotes progression of gastric cancer cells by STAT3-mediated expression of Ror1. Cancer Sci. 2020; 111:1254–65. 10.1111/cas.14339 32012403 PMC7156785

[9] Houghton J, Stoicov C, Nomura S, Rogers AB, Carlson J, Li H, Cai X, Fox JG, Goldenring JR, Wang TC. Gastric cancer originating from bone marrow-derived cells. Science. 2004; 306:1568–71. 10.1126/science.1099513 15567866

[10] Varon C, Dubus P, Mazurier F, Asencio C, Chambonnier L, Ferrand J, Giese A, Senant-Dugot N, Carlotti M, Mégraud F. Helicobacter pylori infection recruits bone marrow-derived cells that participate in gastric preneoplasia in mice. Gastroenterology. 2012; 142:281–91. 10.1053/j.gastro.2011.10.036 22062361

[11] Quante M, Tu SP, Tomita H, Gonda T, Wang SS, Takashi S, Baik GH, Shibata W, Diprete B, Betz KS, Friedman R, Varro A, Tycko B, Wang TC. Bone marrow-derived myofibroblasts contribute to the mesenchymal stem cell niche and promote tumor growth. Cancer Cell. 2011; 19:257–72. 10.1016/j.ccr.2011.01.020 21316604 PMC3060401

[12] von Einem JC, Peter S, Günther C, Volk HD, Grütz G, Salat C, Stoetzer O, Nelson PJ, Michl M, Modest DP, Holch JW, Angele M, Bruns C, et al. Treatment of advanced gastrointestinal cancer with genetically modified autologous mesenchymal stem cells - TREAT-ME-1 - a phase I, first in human, first in class trial. Oncotarget. 2017; 8:80156–66. 10.18632/oncotarget.20964 29113291 PMC5655186

[13] Oya Y, Hayakawa Y, Koike K. Tumor microenvironment in gastric cancers. Cancer Sci. 2020; 111:2696–707. 10.1111/cas.14521 32519436 PMC7419059

[14] Sun C, Dai X, Zhao D, Wang H, Rong X, Huang Q, Lan Q. Mesenchymal stem cells promote glioma neovascularization *in vivo* by fusing with cancer stem cells. BMC Cancer. 2019; 19:1240. 10.1186/s12885-019-6460-0 31864321 PMC6925905

[15] Raz Y, Cohen N, Shani O, Bell RE, Novitskiy SV, Abramovitz L, Levy C, Milyavsky M, Leider-Trejo L, Moses HL, Grisaru D, Erez N. Bone marrow-derived fibroblasts are a functionally distinct stromal cell population in breast cancer. J Exp Med. 2018; 215:3075–93. 10.1084/jem.20180818 30470719 PMC6279405

[16] Yang KQ, Liu Y, Huang QH, Mo N, Zhang QY, Meng QG, Cheng JW. Bone marrow-derived mesenchymal stem cells induced by inflammatory cytokines produce angiogenetic factors and promote prostate cancer growth. BMC Cancer. 2017; 17:878. 10.1186/s12885-017-3879-z 29268703 PMC5740893

[17] Lin R, Ma H, Ding Z, Shi W, Qian W, Song J, Hou X. Bone marrow-derived mesenchymal stem cells favor the immunosuppressive T cells skewing in a Helicobacter pylori model of gastric cancer. Stem Cells Dev. 2013; 22:2836–48. 10.1089/scd.2013.0166 23777268

[18] Nowak-Sliwinska P, Segura T, Iruela-Arispe ML. The chicken chorioallantoic membrane model in biology, medicine and bioengineering. Angiogenesis. 2014; 17:779–804. 10.1007/s10456-014-9440-7 25138280 PMC4583126

[19] Subramanian A, Schilling TF. Thrombospondin-4 controls matrix assembly during development and repair of myotendinous junctions. Elife. 2014; 3:e02372. 10.7554/eLife.02372 24941943 PMC4096842

[20] Lin X, Hu D, Chen G, Shi Y, Zhang H, Wang X, Guo X, Lu L, Black D, Zheng XW, Luo X. Associations of THBS2 and THBS4 polymorphisms to gastric cancer in a Southeast Chinese population. Cancer Genet. 2016; 209:215–22. 10.1016/j.cancergen.2016.04.003 27160021

[21] Muppala S, Frolova E, Xiao R, Krukovets I, Yoon S, Hoppe G, Vasanji A, Plow E, Stenina-Adognravi O. Proangiogenic Properties of Thrombospondin-4. Arterioscler Thromb Vasc Biol. 2015; 35:1975–86. 10.1161/ATVBAHA.115.305912 26139464 PMC4629500

[22] Stenina-Adognravi O, Plow EF. Thrombospondin-4 in tissue remodeling. Matrix Biol. 2019; 75:300–13. 10.1016/j.matbio.2017.11.006 29138119 PMC6005712

[23] Karar J, Maity A. PI3K/AKT/mTOR Pathway in Angiogenesis. Front Mol Neurosci. 2011; 4:51. 10.3389/fnmol.2011.00051 22144946 PMC3228996

[24] Guo D, Zhang D, Ren M, Lu G, Zhang X, He S, Li Y. THBS4 promotes HCC progression by regulating ITGB1 via FAK/PI3K/AKT pathway. FASEB J. 2020; 34:10668–81. 10.1096/fj.202000043R 32567740

[25] Hou Y, Li H, Huo W. THBS4 silencing regulates the cancer stem cell-like properties in prostate cancer via blocking the PI3K/Akt pathway. Prostate. 2020; 80:753–63. 10.1002/pros.23989 32421868

[26] Cingolani OH, Kirk JA, Seo K, Koitabashi N, Lee DI, Ramirez-Correa G, Bedja D, Barth AS, Moens AL, Kass DA. Thrombospondin-4 is required for stretch-mediated contractility augmentation in cardiac muscle. Circ Res. 2011; 109:1410–14. 10.1161/CIRCRESAHA.111.256743 22034490 PMC3324097

[27] Guo X, Oshima H, Kitmura T, Taketo MM, Oshima M. Stromal fibroblasts activated by tumor cells promote angiogenesis in mouse gastric cancer. J Biol Chem. 2008; 283:19864–71. 10.1074/jbc.M800798200 18495668

[28] Borriello L, Nakata R, Sheard MA, Fernandez GE, Sposto R, Malvar J, Blavier L, Shimada H, Asgharzadeh S, Seeger RC, DeClerck YA. Cancer-Associated Fibroblasts Share Characteristics and Protumorigenic Activity with Mesenchymal Stromal Cells. Cancer Res. 2017; 77:5142–57. 10.1158/0008-5472.CAN-16-2586 28687621 PMC5600847

[29] Beckermann BM, Kallifatidis G, Groth A, Frommhold D, Apel A, Mattern J, Salnikov AV, Moldenhauer G, Wagner W, Diehlmann A, Saffrich R, Schubert M, Ho AD, et al. VEGF expression by mesenchymal stem cells contributes to angiogenesis in pancreatic carcinoma. Br J Cancer. 2008; 99:622–31. 10.1038/sj.bjc.6604508 18665180 PMC2527820

[30] Liu Y, Han ZP, Zhang SS, Jing YY, Bu XX, Wang CY, Sun K, Jiang GC, Zhao X, Li R, Gao L, Zhao QD, Wu MC, Wei LX. Effects of inflammatory factors on mesenchymal stem cells and their role in the promotion of tumor angiogenesis in colon cancer. J Biol Chem. 2011; 286:25007–15. 10.1074/jbc.M110.213108 21592963 PMC3137074

[31] Zhu W, Huang L, Li Y, Zhang X, Gu J, Yan Y, Xu X, Wang M, Qian H, Xu W. Exosomes derived from human bone marrow mesenchymal stem cells promote tumor growth *in vivo*. Cancer Lett. 2012; 315:28–37. 10.1016/j.canlet.2011.10.002 22055459

[32] Stenina OI, Desai SY, Krukovets I, Kight K, Janigro D, Topol EJ, Plow EF. Thrombospondin-4 and its variants: expression and differential effects on endothelial cells. Circulation. 2003; 108:1514–19. 10.1161/01.CIR.0000089085.76320.4E 12952849

[33] Frolova EG, Pluskota E, Krukovets I, Burke T, Drumm C, Smith JD, Blech L, Febbraio M, Bornstein P, Plow EF, Stenina OI. Thrombospondin-4 regulates vascular inflammation and atherogenesis. Circ Res. 2010; 107:1313–25. 10.1161/CIRCRESAHA.110.232371 20884877 PMC2993182

[34] Förster S, Gretschel S, Jöns T, Yashiro M, Kemmner W. THBS4, a novel stromal molecule of diffuse-type gastric adenocarcinomas, identified by transcriptome-wide expression profiling. Mod Pathol. 2011; 24:1390–403. 10.1038/modpathol.2011.99 21701537

[35] McCart Reed AE, Song S, Kutasovic JR, Reid LE, Valle JM, Vargas AC, Smart CE, Simpson PT. Thrombospondin-4 expression is activated during the stromal response to invasive breast cancer. Virchows Arch. 2013; 463:535–45. 10.1007/s00428-013-1468-3 23942617

[36] Kuroda K, Yashiro M, Sera T, Yamamoto Y, Kushitani Y, Sugimoto A, Kushiyama S, Nishimura S, Togano S, Okuno T, Tamura T, Toyokawa T, Tanaka H, et al. The clinicopathological significance of Thrombospondin-4 expression in the tumor microenvironment of gastric cancer. PLoS One. 2019; 14:e0224727. 10.1371/journal.pone.0224727 31703077 PMC6839882

[37] Chen X, Huang Y, Wang Y, Wu Q, Hong S, Huang Z. THBS4 predicts poor outcomes and promotes proliferation and metastasis in gastric cancer. J Physiol Biochem. 2019; 75:117–23. 10.1007/s13105-019-00665-9 30746617

[38] Muppala S, Xiao R, Krukovets I, Verbovetsky D, Yendamuri R, Habib N, Raman P, Plow E, Stenina-Adognravi O. Thrombospondin-4 mediates TGF-β-induced angiogenesis. Oncogene. 2017; 36:5189–98. 10.1038/onc.2017.140 28481870 PMC5589494

[39] Frolova EG, Sopko N, Blech L, Popovic ZB, Li J, Vasanji A, Drumm C, Krukovets I, Jain MK, Penn MS, Plow EF, Stenina OI. Thrombospondin-4 regulates fibrosis and remodeling of the myocardium in response to pressure overload. FASEB J. 2012; 26:2363–73. 10.1096/fj.11-190728 22362893 PMC3360147

[40] Pluskota E, Stenina OI, Krukovets I, Szpak D, Topol EJ, Plow EF. Mechanism and effect of thrombospondin-4 polymorphisms on neutrophil function. Blood. 2005; 106:3970–78. 10.1182/blood-2005-03-1292 16099885 PMC1895095

[41] Brody MJ, Vanhoutte D, Schips TG, Boyer JG, Bakshi CV, Sargent MA, York AJ, Molkentin JD. Defective Flux of Thrombospondin-4 through the Secretory Pathway Impairs Cardiomyocyte Membrane Stability and Causes Cardiomyopathy. Mol Cell Biol. 2018; 38:e00114–18. 10.1128/MCB.00114-18 29712757 PMC6024163

[42] Graupera M, Guillermet-Guibert J, Foukas LC, Phng LK, Cain RJ, Salpekar A, Pearce W, Meek S, Millan J, Cutillas PR, Smith AJ, Ridley AJ, Ruhrberg C, et al. Angiogenesis selectively requires the p110alpha isoform of PI3K to control endothelial cell migration. Nature. 2008; 453:662–66. 10.1038/nature06892 18449193

[43] Phung TL, Ziv K, Dabydeen D, Eyiah-Mensah G, Riveros M, Perruzzi C, Sun J, Monahan-Earley RA, Shiojima I, Nagy JA, Lin MI, Walsh K, Dvorak AM, et al. Pathological angiogenesis is induced by sustained Akt signaling and inhibited by rapamycin. Cancer Cell. 2006; 10:159–70. 10.1016/j.ccr.2006.07.003 16904613 PMC2531257

