# Supplementary material for: THBS4/integrin α2 axis mediates BM-MSCs to promote angiogenesis in gastric cancer associated with chronic Helicobacter pylori infection
Source: Aging (Albany NY). 2021 Aug 14;13(15):19375–96. doi: 10.18632/aging.203334 (PMC8386559; doi:10.18632/aging.203334)
Supplement: Supplementary Figures [file aging-13-203334-s001.pdf]

SUPPLEMENTARY FIGURES

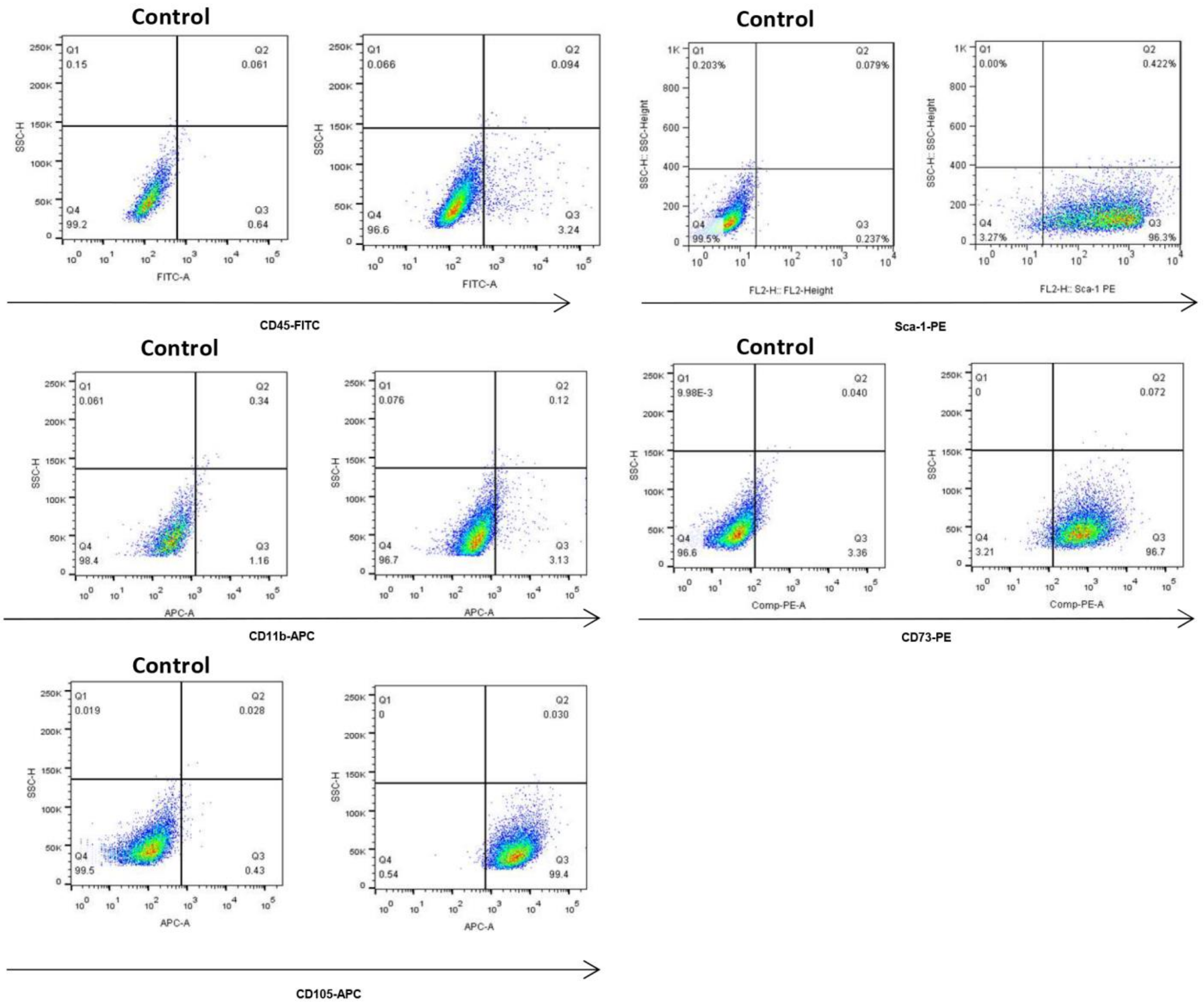

Supplementary Figure 1. Flow cytometry for cell surface antigens of BM-MSCs. The left columns were control BM-MSCs, which were not incubated with antibodies. The right columns were BM-MSCs incubated with antibodies.

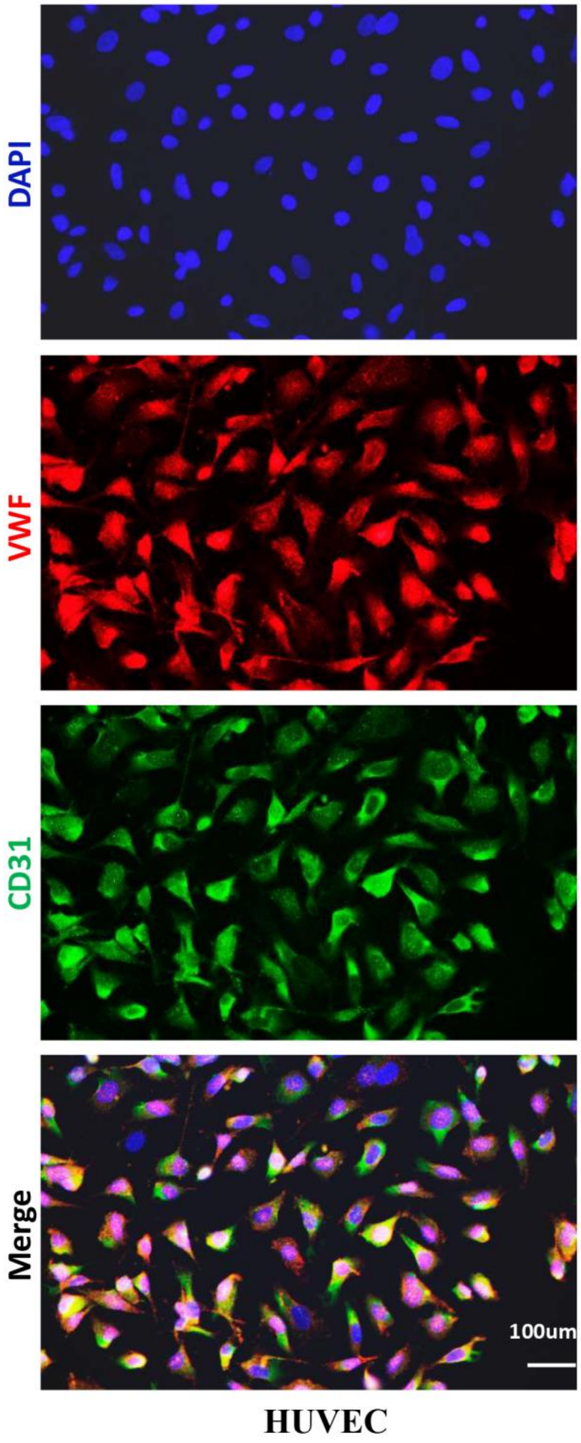

Supplementary Figure 2. Immunofluorescence staining of VWF and CD31 for cell surface antigens of HUVECs.
