# Supplementary material for: THBS4/integrin α2 axis mediates BM-MSCs to promote angiogenesis in gastric cancer associated with chronic Helicobacter pylori infection
Source: Aging (Albany NY). 2021 Aug 14;13(15):19375–96. doi: 10.18632/aging.203334 (PMC8386559; doi:10.18632/aging.203334)
Supplement: Supplementary Table 1 [file aging-13-203334-s002.pdf]

SUPPLEMENTARY TABLE

Supplementary Table 1. Primers for qRT-PCR.

Number	Primer for qRT-PCR (mouse)	Forward primer	Reverse primer
1	THBS4	AGAGTTCCCAACCGTGACCA	AGGAGTCCCCAACCAGATCG
2	Myl3	AGCCAGAGCCCAAGAAGGAT	TTGGAGGCATCAAACTCGGC
3	Kng1	ACTGTAAGTCCACCCTACATTG	CCACGGTGATTCTTATTAGCCT
4	Prodh2	CCTCAGCACAGAACAGAATCAG	GACAGTGCAGGGTTAATGAAAG
5	Serpina1d	AAACAGGCGCAGAAGCGATG	GAGAGGTCAGCCCCATTGTT
6	Angptl3	GATGACCTTCCTGCCGACTG	TGGACTGCCTGATTGGGTATC
